# 

**Published:** 1859-03

**Authors:** 


					﻿The Medical Society of Georgia, will convene in
Annual Session, in the city of Atlanta, on the second
Wednesday in April, 1859. The members of the Society,
and those feeling an interest, in the advancement of Medi-
cal Science in the State, are earnestly invited to attend.
SUBSCRIPTIONS RECEIVED.
R. J. Bigelow, Fla., $3; A. B. Wallace, Ga., $3; Middle-
brooks, $3; B. F. Bomar, Ga., $7.50; J. R. Vaughan, Ala.,
$3; W. B. Couch, Ga., $2 ; B. C. Biggers, Ala., $2 ; Willis
Willingham, Ga., $6; W. P. Anderson, Ga., $6; ----Beas-
ley, Ga., $2.
TO CORRESPONDENTS.
fgf All Cemmunications pertaining tv the Editorial Department of the Journal,
should be addressed to the Editors.
flgy- All Communications, having reference to the Subscription, the Advertising
or the Financial Lej ailment, tl.culd le addressed to the Tieasurer, J. G. West-
)1< beland, M. D., Dean of the Faculty of the Atlanta Medical College.
				

